# Landscapes of gut bacterial and fecal metabolic signatures and their relationship in severe preeclampsia

**DOI:** 10.1186/s12967-024-05143-5

**Published:** 2024-04-17

**Authors:** Xianxian Liu, Xiaoming Zeng, Xing Li, Siming Xin, Feng Zhang, Faying Liu, Yang Zeng, Jilin Wu, Yang Zou, Xinwei Xiong

**Affiliations:** 1 Key Laboratory of Women’s Reproductive Health of Jiangxi Province, Jiangxi Maternal and Child Health Hospital, Nanchang, Jiangxi 330006 China; 2 Institute of Biological Technology, Nanchang Normal University, Nanchang, Jiangxi 330032 China; 3 Medical Center of Burn Plastic and Wound Repair, The First Affiliated Hospital of Nanchang University, Jiangxi Medical College, Nanchang University, Nanchang, 330006 China

**Keywords:** Severe preeclampsia, Gut microbiome, Metagenomic sequencing, Fecal metabolome, Biomarkers

## Abstract

**Background:**

Preeclampsia is a pregnancy-specific disease leading to maternal and perinatal morbidity. Hypertension and inflammation are the main characteristics of preeclampsia. Many factors can lead to hypertension and inflammation, including gut microbiota which plays an important role in hypertension and inflammation in humans. However, alterations to the gut microbiome and fecal metabolome, and their relationships in severe preeclampsia are not well known. This study aims to identify biomarkers significantly associated with severe preeclampsia and provide a knowledge base for treatments regulating the gut microbiome.

**Methods:**

In this study, fecal samples were collected from individuals with severe preeclampsia and healthy controls for shotgun metagenomic sequencing to evaluate changes in gut microbiota composition. Quantitative polymerase chain reaction analysis was used to validate the reliability of our shotgun metagenomic sequencing results. Additionally, untargeted metabolomics analysis was performed to measure fecal metabolome concentrations.

**Results:**

We identified several Lactobacillaceae that were significantly enriched in the gut of healthy controls, including *Limosilactobacillus fermentum*, the key biomarker distinguishing severe preeclampsia from healthy controls. *Limosilactobacillus fermentum* was significantly associated with shifts in KEGG Orthology (KO) genes and KEGG pathways of the gut microbiome in severe preeclampsia, such as flagellar assembly. Untargeted fecal metabolome analysis found that severe preeclampsia had higher concentrations of Phenylpropanoate and Agmatine. Increased concentrations of Phenylpropanoate and Agmatine were associated with the abundance of *Limosilactobacillus fermentum*. Furthermore, all metabolites with higher abundances in healthy controls were enriched in the arginine and proline metabolism pathway.

**Conclusion:**

Our research indicates that changes in metabolites, possibly due to the gut microbe *Limosilactobacillus fermentum*, can contribute to the development of severe preeclampsia. This study provides insights into the interaction between gut microbiome and fecal metabolites and offers a basis for improving severe preeclampsia by modulating the gut microbiome.

**Supplementary Information:**

The online version contains supplementary material available at 10.1186/s12967-024-05143-5.

## Background

Preeclampsia is a gestational multisystem disease characterized by hypertension and significant proteinuria after 20 weeks of pregnancy [1, 2]. Globally, the incidence of preeclampsia among pregnant women is approximately 2–5%, resulting in more than 500,000 infant deaths and 76,000 maternal deaths annually [3]. This condition has substantial adverse effects on both the mother and the fetus. For the mother, preeclampsia can induce coagulation and hepatorenal impairment [4, 5]. In the case of the fetus, it leads to growth restriction and placental abruption, increasing the risk of premature birth or stillbirth [6]. Numerous theories have been proposed to explain the pathogenesis of preeclampsia in pregnant women, encompassing factors such as hypertension, inflammation, and dysfunction [7, 8]. These theories collectively suggest the involvement of dysbiosis in the gut microbiota in the development of preeclampsia.

In recent years, numerous studies have unveiled connections between the composition of gut microbiota and human diseases. The available evidence suggests that interference with the balance of “normal” gut microbiota, also known as enteric dysbiosis might contribute to the development of various health conditions, such as hypertension and preeclampsia. The gut microbiota of hypertensive patients is significantly abnormal, and addressing gut microbiota dysbiosis can effectively reduce blood pressure [9, 10]. This conclusion had also been observed in high-salt-induced hypertensive mice. Wilck et al. discovered that *Lactobacillus murinus* inhibits the differentiation of T lymphocytes into Th17 cells by producing indole compounds, thereby reducing blood pressure and inflammation [11]. Yan et al. demonstrated that *Bacteroides fragile* could lower blood pressure by antagonizing the production of intestinal corticosterone [12]. Several studies have shown that the gut microbiota of preeclamptic patients is markedly abnormal [13–15]. Brantsaeter et al. found that a high intake of probiotics containing lactobacilli can mitigate the risk of preeclampsia [16]. Furthermore, preeclampsia is linked to dysbiosis in the intestinal microbiota and the presence of short-chain fatty acids derived from the intestine. In a study by Jin et al., it was shown that *Akkermansia muciniphila* and propionate/butyrate exhibit significant potential for treating preeclampsia [17]. However, the relationship between dysbiosis in gut microbiota and the pathogenesis of preeclampsia remains unclear.

In this study, fecal samples were collected from pregnant women exhibiting severe preeclampsia and from healthy individuals in the same trimester. We first performed shotgun metagenomic sequencing analyses on fecal samples to identity gut bacterial taxa and function capacities associated with severe preeclampsia. We then measured the concentrations of fecal metabolites by untargeted metabolome methods to identify the metabolites associated with severe preeclampsia and analyze its correlations with the shifts of gut microbiome. The objective of this research is to identify biomarkers significantly associated with severe preeclampsia, ultimately contributing to our understanding of this condition and offering insights into potential interventions for its improvement through the regulation of the gut microbiome.

## Materials and methods

### Sample collection

This single-center retrospective study included 198 severe preeclampsia patients, admitted to the Jiangxi Maternal and Child Health Hospital between 1 January 2021 and 31 December 2023. Pregnant women with severe preeclampsia were classified according to the International Society for the Study of Hypertension in Pregnancy (ISSHP). Diagnosis of severe preeclampsia was based on increased systolic or diastolic blood pressure, accompanied by proteinuria and one or more of the conditions at ≥ 20 weeks of gestation as described in previous studies [18, 19]. For example: (1) systolic blood pressure (SBP) ≥ 160 mmHg and/or diastolic blood pressure (DBP) ≥ 110 mmHg on two separate measurements at least 4 h apart during bed rest; (2) serum alanine aminotransferase (ALT) or aspartate aminotransferase (AST) levels more than 2 times the upper limit of the normal range. In addition, 400 controls were also recruited in the same period. No hypertension/preeclampsia/severe preeclampsia was found in these controls until delivery. Exclusion criteria for both groups included: (1) Congenital anomalies of kidneys and kidney vessels, chronic kidney diseases, asymptomatic bacteriuria or pyelonephritis, and ischemic heart disease; (2) pregnancy complications such as diabetes mellitus, chronic hypertension, intrahepatic cholestasis of pregnancy, autoimmune conditions (e.g., thyroid dysfunction, systemic lupus erythematosus, and anticardiolipin syndrome). Furthermore, to investigate the differences in the gut bacterial and fecal metabolic profiles between severe preeclampsia and healthy controls, 18 individuals of similar gestational weeks and age were selected from the two groups within two months. Fresh feces from 18 individuals were collected from their anus through anal examination after the cesarean section. An overview flow chart of this study is shown in Fig. S1 (Additional file 1). The primary endpoints of this study were the identification of bacterial species and metabolites associated with severe preeclampsia, and relationships among bacterial species, metabolites and clinal data. Secondary endpoints were to identify and confirm the biomarkers associated with severe preeclampsia to improve or predict severe preeclampsia.

### Metagenome DNA extraction and shotgun sequencing

The genomic DNA samples from the microbial community were extracted utilizing the OMEGA Mag-Bind Soil DNA Kit (M5635-02) (Omega Bio-Tek, Norcross, GA, USA) in accordance with the manufacturer’s guidelines. Subsequently, these samples were stored at -20 °C for subsequent analysis. The quantification and assessment of DNA quality were performed using agarose gel electrophoresis and a Qubit™ 4 Fluorometer (Invitrogen, USA), respectively. To construct metagenome shotgun sequencing libraries, the extracted microbial DNA underwent processing, with insert sizes targeted at 400 bp, utilizing the Illumina TruSeq Nano DNA LT Library Preparation Kit. Each resulting library was sequenced on the Illumina NovaSeq platform (Illumina, USA) employing the PE150 strategy.

### Metagenomics analysis

The raw sequencing reads underwent processing to acquire quality-filtered reads for subsequent analysis. Initially, sequencing adapters were eliminated from the reads using Cutadapt (v1.2.1) [20]. Subsequently, a sliding-window algorithm in fastp was employed to trim low-quality reads [21]. Next, the reads were aligned to the human reference genome (Homo sapiens GRCh38.p12) using BMTagger to eliminate host contamination [22]. Following the acquisition of quality-filtered reads, taxonomical classifications of metagenomic sequencing reads from each sample were conducted using Kraken2 [23] against a RefSeq-derived database, encompassing bacterial genomes. Megahit (v1.1.2) [24] was utilized to assemble each sample with the meta-large preset parameters. The contigs (length exceeding 300 bp) were pooled and clustered using mmseqs2 [25] with “easy-linclust”. MetaGeneMark [26] was used to predict the genes in the contigs. CDS sequences of all samples were clustered by mmseqs2 [25] with “easy-cluster” mode, setting the protein sequence identity threshold to 0.90 and covering residues of the shorter contig to 90%. To evaluate the abundance of these genes, high-quality reads from each sample were mapped onto the predicted gene sequences using salmon [27]. The copy per kilobase per million mapped reads was employed to normalize abundance values in metagenomes. The functionality of the non-redundant genes was determined by annotation using mmseqs2 [25] with the “search” mode against the protein databases of KEGG.

### Determination of metabolomic profiles of fecal samples

A total of 18 fecal samples were utilized for untargeted metabolomic analysis through LC-MS/MS. The methods for executing LC-MS/MS are described in previous studies [28, 29]. Briefly, an appropriate amount of each sample was accurately weighed into a 2 mL centrifuge tube. Subsequently, 600 µL of MeOH (containing 2-Amino-3-(2-chloro-phenyl)-propionic acid at 4 ppm) was added, followed by vortexing for 30 s. The tissue grinder was then loaded with steel balls and operated at 50 Hz for 120 s, after which ultrasound was applied for 10 min at room temperature. The resulting mixture underwent centrifugation for 10 min at 12,000 rpm and 4 ℃. The supernatant was using a 0.22 μm membrane and transferred into the detection bottle for LC-MS detection. The LC analysis was performed on a Vanquish UHPLC System (Thermo Fisher Scientific, USA). Mass spectrometric detection of metabolites was performed on Q Exactive Focus (Thermo Fisher Scientific, USA) with an ESI ion source. Simultaneous MS1 and MS/MS (Full MS-ddMS2 mode, data-dependent MS/MS) acquisition was used. We used a mzXML format by utilizing MSConvert in the ProteoWizard software package (v3.0.8789) [30] to convert the raw data and XCMS [31] to process for feature detection, retention time correction and alignment, respectively. After normalization, only ion peaks with relative standard deviations (RSDs) less than 30% in QC were kept to ensure proper metabolite identification.

### Quantitative polymerase chain reaction (qPCR) assay

The abundance of *Limosilactobacillus fermentum* was further confirmed through the quantification of 16S gene copies in 12 fecal DNA samples using qPCR following a random selection from the metagenomic sequencing samples. The qPCR reactions were carried out with the primer set 16S-F (5’- CGTAGGTGGCAAGCGTTATC-3’)/16S-R (5’-CATTCCACCGCTACACATGG-3’). A 20 µL reaction volume included 10 µL of 2× SYBR real-time PCR premixture (Vazyme, China), 2 µL of template and 0.4 µL each of forward and reverse primers (10 μm each), plated in 384-well plates. The qPCR was executed on a LightCycler480II real-time System (Roche, Switzerland) with the following thermal conditions: 5 min at 95℃, followed by 40 cycles of 15 s at 95 ℃ and 30 s at 60 ℃. To generate a standard curve eight 10-fold diluted plasmids carrying the *16 S* gene were employed (Additional file 2: Fig. S2). Each qPCR sample underwent triplicate analysis, ensuring that three replicates were utilized for each assessment.

### Statistical analysis

To identify differences in abundances between the two groups, a linear discriminant analysis (LDA) effect size (LEfSe) analysis was conducted with a significance threshold of α = 0.05 and an LDA score of at least 2.0 [32]. The FDR correction, using Story’s method, was applied to account for multiple tests. Additionally, Partial Least Squares-Discriminant Analysis (PLS-DA) was employed to assess fecal metabolite profiles within the untargeted metabolome, comparing severe preeclampsia with healthy controls [33].

## Results

### Clinical characteristics of the pregnant women

The study involved the phenotyping of 598 pregnant women, encompassing 14 clinical characteristics. Among these, 198 pregnant women exhibited severe preeclampsia, while the remaining 400 were healthy controls within the same trimesters. A comprehensive overview of the statistical outcomes pertaining to the 14 clinical characteristics is provided in Table 1. The results revealed significant differences in the majority of the tested clinical characteristics, as indicated by the t-test with a significance level of *P* < 0.05. Specifically, in comparison to women with severe preeclampsia, healthy pregnant women exhibited significantly higher birth weight (t-test, *P* < 0.001) and Apgar score (t-test, *P* < 0.001) at the time of sampling (Table 1). Contrastingly, the age of participants did not exhibit a significant difference between those with severe preeclampsia and those with normal pregnancies (t-test, *P* > 0.05) (Table 1).

**Table 1:** The statistical outcomes pertaining to the 14 clinical characteristics

Traits	Normal Group^1^ (*n* = 400)	Severe preeclampsia Group^1^ (*n* = 198)	P-value
Age	30.14 ± 4.51	30.48 ± 4.66	0.34
Body weight	68.15 ± 8.12	71.44 ± 8.91	2.17E-04
BMI	26.94 ± 3.10	28.30 ± 3.28	1.88E-06
SP	119.77 ± 10.46	148.91 ± 17.81	1.44E-93
DP	72.45 ± 8.02	93.95 ± 14.01	1.02E-86
Birth weight	3.17 ± 0.32	2.31 ± 0.89	1.53E-51
Apgar score	9.71 ± 0.60	8.92 ± 1.41	5.60E-19
PLT	213.24 ± 55.47	202.05 ± 72.74	3.09E-02
ALT	10.71 ± 5.31	17.74 ± 19.56	2.87E-11
AST	18.90 ± 4.91	28.01 ± 21.40	2.53E-15
TP	62.66 ± 5.14	58.23 ± 6.84	2.02E-17
ALB	34.22 ± 3.51	30.94 ± 4.29	4.70E-21
CREA	45.88 ± 8.92	64.82 ± 24.40	1.93E-36
Cys-C	0.97 ± 0.16	1.25 ± 0.49	5.00E-19

^1^Mean ± Standard deviation

### Identification of bacterial species associated with severe preeclampsia

To further identify bacterial species and assess the functional capacity of the gut microbiome associated with severe preeclampsia, we randomly selected and conducted shotgun metagenomic sequencing on 18 fecal samples. These samples comprised ten patients with severe preeclampsia and eight healthy controls from the same trimester. The sequence assembly of the 18 samples yielded a total of 2,618,386 sequences, with an average length of 1,949 bp and an average N50 length of 6,461 bp (Additional file 2: Table S1). The phylogenetic composition of the fecal microbiota was determined by blasting the contigs against the NCBI nucleotide (NR) database. The two most abundant genera of gut microbiota were *Bacteroides* and *Faecalibacterium*. At the species level, we detected a total of 3,141 bacterial species in the 18 samples. *Gemmiger qucibialis* was found to be the most abundant bacterial species among the tested samples.

**Fig. 1:**
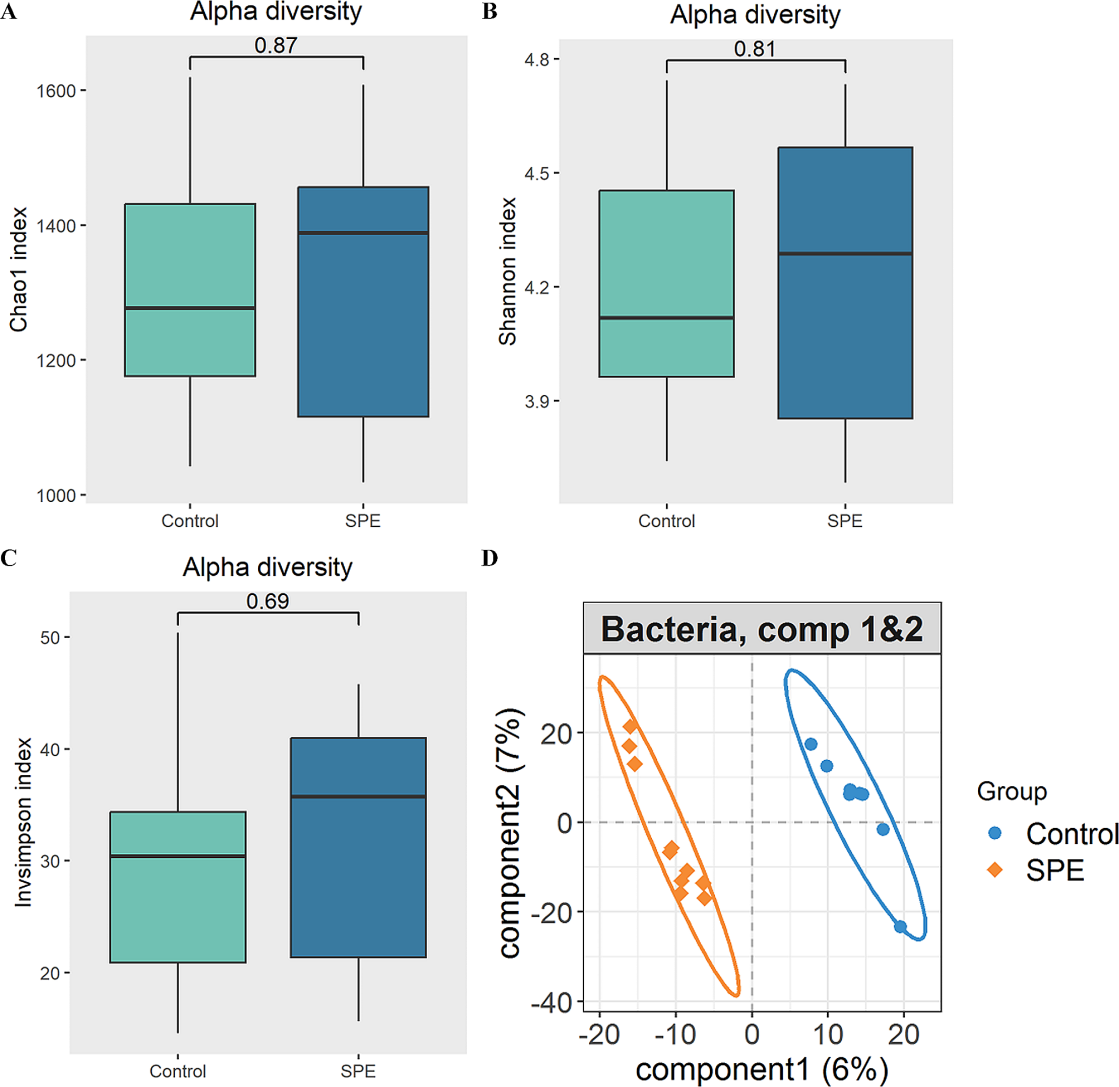
Gut microbiome characteristics in severe preeclampsia versus healthy controls. **A-C** There were no significant bacterial α diversity differences between the two groups. **D** PLS-DA plot of fecal bacterial species, which indicates the substantial differentiation in gut bacterial species between the two groups. SPE: severe preeclampsia; Control: healthy controls

**Fig. 2:**
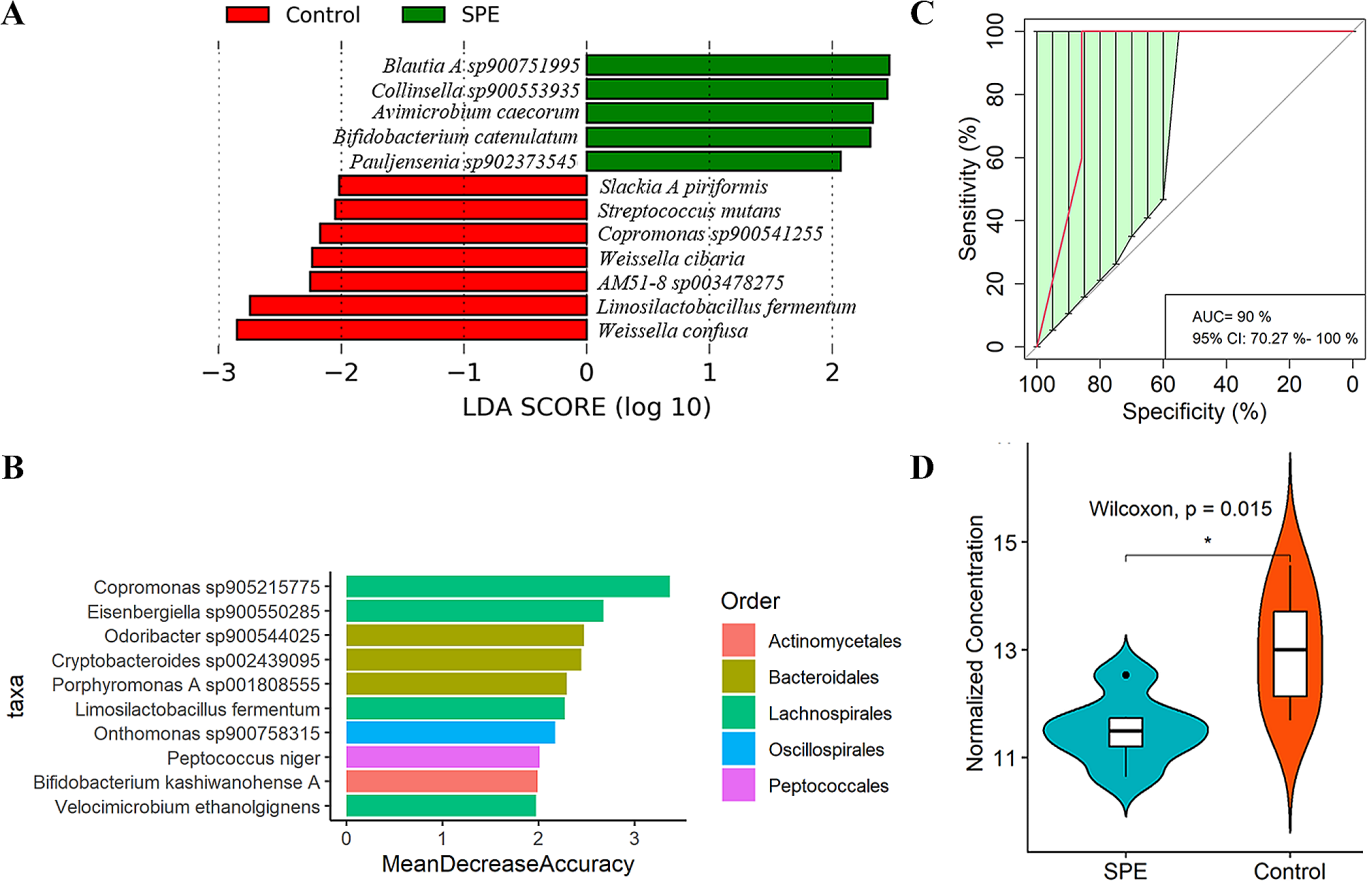
Identification of bacterial species associated with severe preeclampsia based on metagenomic sequencing data. **A** Bacterial species showing significantly different abundances between severe preeclampsia and healthy controls. LEfSe analysis was used to identify differential bacterial species. LDA score ≥ 2 was set as the threshold. **B** Bacterial species that could discriminate severe preeclampsia and healthy controls by Random Forest model. **C** Receiver operating curve (ROC). The AUC was 90.00% with a 95% CI of 70.27–100%. **D** The abundance of *Limosilactobacillus fermentum* was confirmed by qPCR. SPE: severe preeclampsia; Control: healthy controls

We conducted a comparative analysis of the gut microbiome bacterial composition between individuals with severe preeclampsia and healthy controls to identify specific bacterial species associated with severe preeclampsia. Initially, the bacterial α-diversity analysis revealed no significant differences in the Chao, Shannon, and Invsimpson indexes between individuals with severe preeclampsia and healthy controls (Fig. 1A-C). Subsequently, we investigated whether there were differences in the overall bacterial species between the two groups. PLS-DA demonstrated a distinct shift in bacterial species distribution (Fig. 1D). Moreover, our analysis identified 12 bacterial species with varying abundances between the severe preeclampsia and healthy control groups (Fig. 2A). Among these, five species were significantly enriched in severe preeclampsia, while seven species exhibited higher abundance in healthy pregnant women. Notably, the bacteria enriched in the gut of healthy pregnant women predominantly belonged to the Lactobacillaceae (3 out of 7 species). Subsequently, a random forest analysis was conducted to identify bacterial biomarkers that could discriminate severe preeclampsia from healthy pregnant women based on metagenomic sequencing data at the species level (Fig. 2B). Interestingly, consistent with the findings in the LEfSe analysis, *Limosilactobacillus fermentum* emerged as the sole bacterial species that exhibited significant differences between severe preeclampsia and healthy controls. Notably, *Limosilactobacillus fermentum* demonstrated robust and high diagnostic accuracy, with an area under the curve (AUC) of 90.00% (Fig. 2C).

To further validate the results of the metagenomic sequencing, the abundance of *Limosilactobacillus fermentum* was quantified using qPCR. Twelve samples (six from each group), randomly selected from those subjected to metagenomic sequencing, were utilized for qPCR analysis. The results indicated that, in comparison to the healthy pregnant women group, individuals of *Limosilactobacillus fermentum* in the severe preeclampsia group exhibited significantly lower abundance (*P* < 0.05, FDR), thereby affirming the high reproducibility of the metagenomic sequencing results (Fig. 2D).

### Identification of microbial function capacity associated with severe preeclampsia

**Fig. 3:**
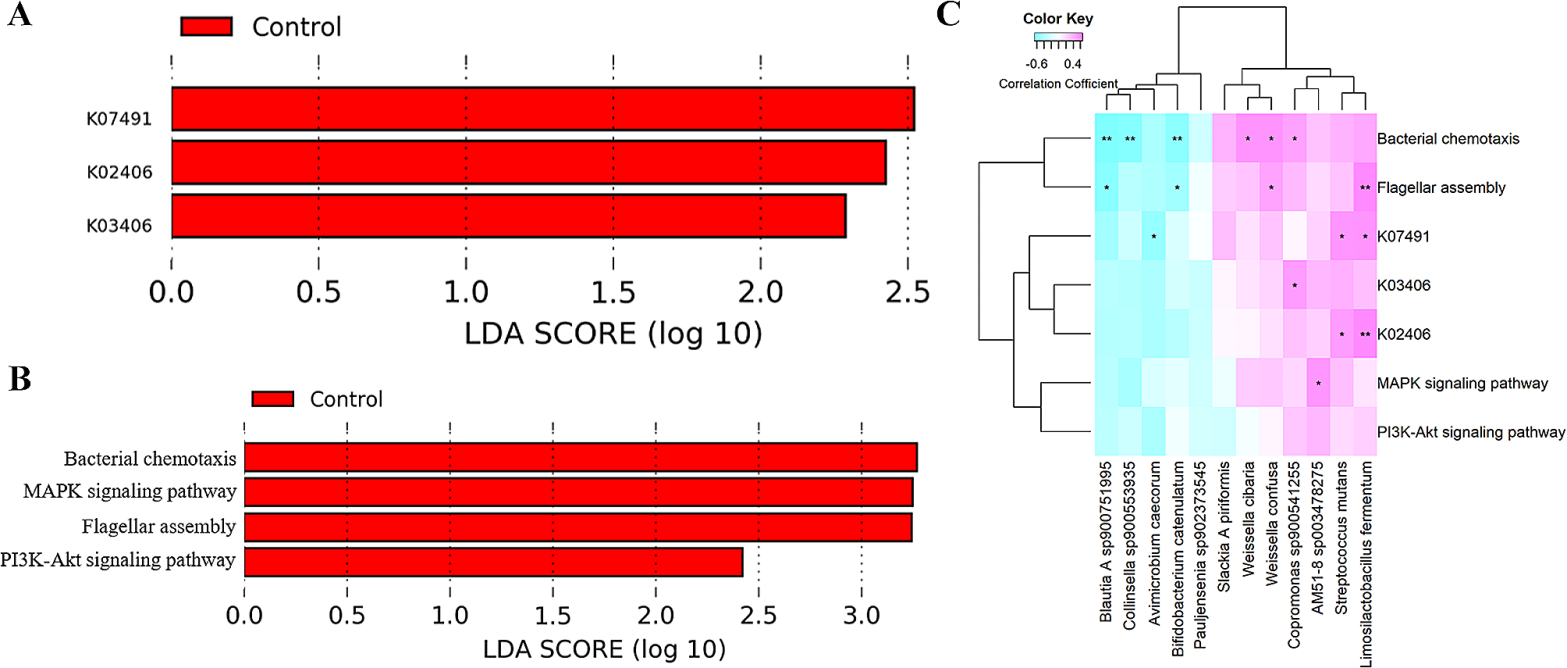
KEGG pathways and KO genes show significant shifts in abundances between severe preeclampsia and healthy controls, and their relationships with differential bacterial species. **A** Differential KO genes. **B** Differential KEGG pathways. **C** The heat maps showing the relationships between differential bacterial species, and differential KEGG pathways and KO genes. The *X*-axis represents the differential bacterial species. The *Y*-axis indicates the differential KEGG pathways and KO genes. * *P* < 0.05, ** *P* < 0.01, and *** *P* < 0.001. SPE: severe preeclampsia; Control: healthy controls

We then compared the potential functional capacities of the gut microbiome between severe preeclampsia and healthy controls by aligning and classifying the microbial genes to the KEGG databases. Three KEGG Orthology (KO) genes (Fig. 3A) and four KEGG pathways (Fig. 3B) with distinct enrichments were identified between severe preeclampsia and healthy controls, respectively. The three different KO genes K07491 (REP-associated tyrosine transposase), K02406 (flagellin), and K03406 (methyl-accepting chemotaxis protein) were enriched in healthy pregnant women. Similarly, the four different KEGG pathways bacterial chemotaxis, MAPK signaling pathway, flagellar assembly, and PI3K-Akt signaling pathway also exhibited higher abundances in healthy pregnant women. Notably, K02406 (flagellin) and K03406 (methyl-accepting chemotaxis protein) were related to the KEGG pathways flagellar assembly and bacterial chemotaxis, respectively. We further assessed the correlation between shifts in bacterial species and changes in KO genes and KEGG pathways using Spearman correlation analysis. The results indicated that *Limosilactobacillus fermentum* was positively and significantly correlated with K02406 (flagellin), K07491 (REP-associated tyrosine transposase) and flagellar assembly (Fig. 3C). These findings suggest that changes in bacterial species contribute to shifts in KO genes and KEGG pathways.

### Differential fecal metabolite profiles between severe preeclampsia and healthy controls

**Fig. 4:**
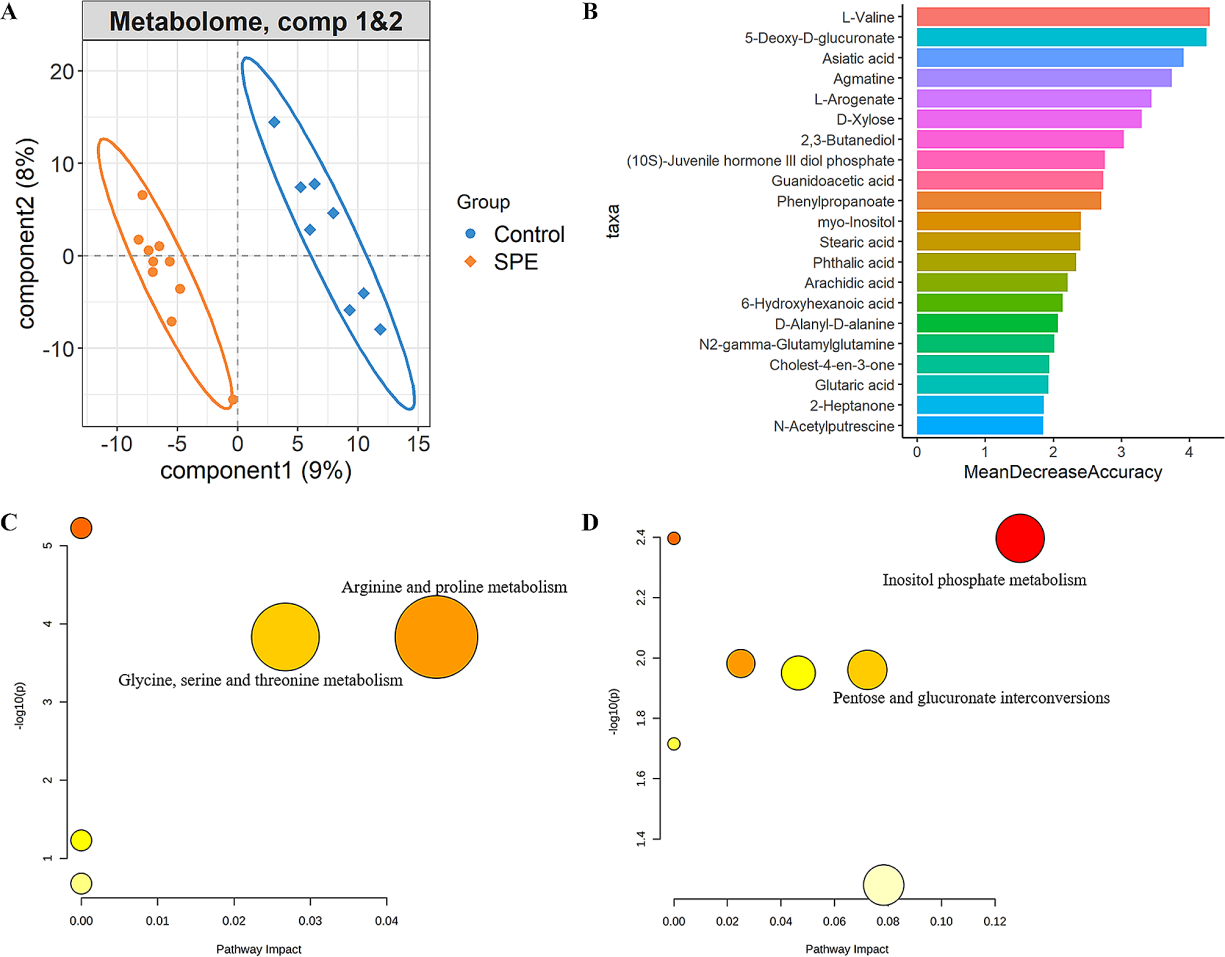
The changes in fecal metabolome between severe preeclampsia and healthy controls, and functional enrichment of differential fecal metabolite features. **A** PLS-DA plot of fecal metabolite profiles indicates the significant differentiation of fecal metabolite profiles between severe preeclampsia and healthy controls. **B** Significantly different abundances of fecal metabolites by Random Forest model. **C**-**D** KEGG pathways enriched by the metabolite features show higher abundances in f healthy controls (**C** ) and severe preeclampsia (**D** ). The *X*-axis and the size of the dots indicate the pathway impact of altered metabolite features, and the *Y*-axis shows the *P*-value obtained for each pathway in the enrichment analysis. The size and color of the dots indicate the overall pathway impact. SPE: severe preeclampsia; Control: healthy controls

To systematically evaluate the shifts of fecal metabolome between severe preeclampsia and healthy controls, untargeted fecal metabolomic profiles were determined using LC-MS/MS. A total of 686 fecal metabolites were obtained after normalization. An obvious shift in the fecal metabolome was observed between severe preeclampsia and healthy controls (Fig. 4A). Specifically, we identified a total of 31 metabolite features showing distinct enrichment patterns between severe preeclampsia and healthy controls (Wilcoxon, *P* < 0.05; Additional file 3: Table S2). Then, a random forest analysis and LASSO regression analysis were performed to identify fecal biomarkers for discriminating severe preeclampsia from healthy pregnant women based on fecal metabolomic profiles. A total of 21 (Fig. 4B) and 17 (Additional file 4: Table S3) metabolite features were identified as differential metabolites between severe preeclampsia and healthy controls, respectively. Interestingly, six differential metabolite features were identified in all three analyses. These enriched metabolites included two metabolite features that were significantly enriched in healthy pregnant women, and four metabolite features that were significantly enriched in severe preeclampsia. For example, Guanidoacetic acid and L-Valine, 5-Deoxy-D-glucuronate, Phenylpropanoate, Agmatine, and N-Acetylputrescine were enriched in healthy pregnant women and severe preeclampsia, respectively. The differential metabolite features identified in severe preeclampsia and healthy controls were separately annotated and classified using KEGG pathways. We found that the metabolites having higher abundances in healthy controls were significantly enriched in the KEGG pathway of arginine and proline metabolism and glycine, serine and threonine metabolism (Fig. 4C). In comparison, the pathway of inositol phosphate metabolism and pentose and glucuronate interconversions were enriched by the metabolites having higher abundances in severe preeclampsia (Fig. 4D).

**Fig. 5:**
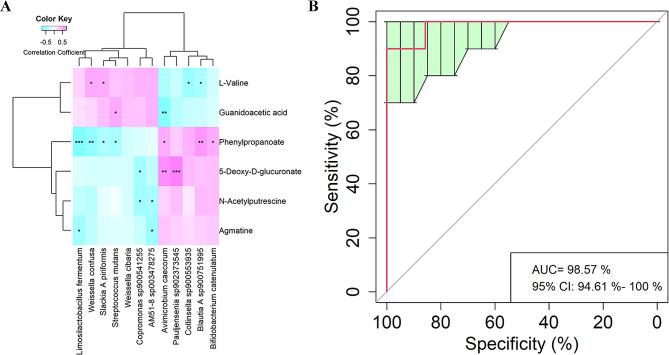
The relationships of altered fecal metabolite features with differential bacterial species, and Receiver operating curve (ROC). **A** Relationships. The *X*-axis represents differential fecal metabolite features. The *Y*-axis represents the differential bacterial species. * *P* < 0.05, ** *P* < 0.01, and *** *P* < 0.001. **B** ROC. The AUC was 98.57% with a 95% CI of 94.61–100%

A Spearman correlation analysis was performed to further evaluate the correlations between differential bacterial species and metabolite features. The results indicate that *Limosilactobacillus fermentum* was negatively and significantly correlated with Phenylpropanoate (*P* < 0.001) and Agmatine (*P* < 0.05), respectively (Fig. 5A). Furthermore, Phenylpropanoate could be used to distinguish severe preeclampsia and healthy pregnant women with robust and high diagnostic accuracy of the area under the curve (AUC) 98.57% (Fig. 5B).

### Co‑occurrence analysis between the gut bacteria and fecal metabolites

**Fig. 6:**
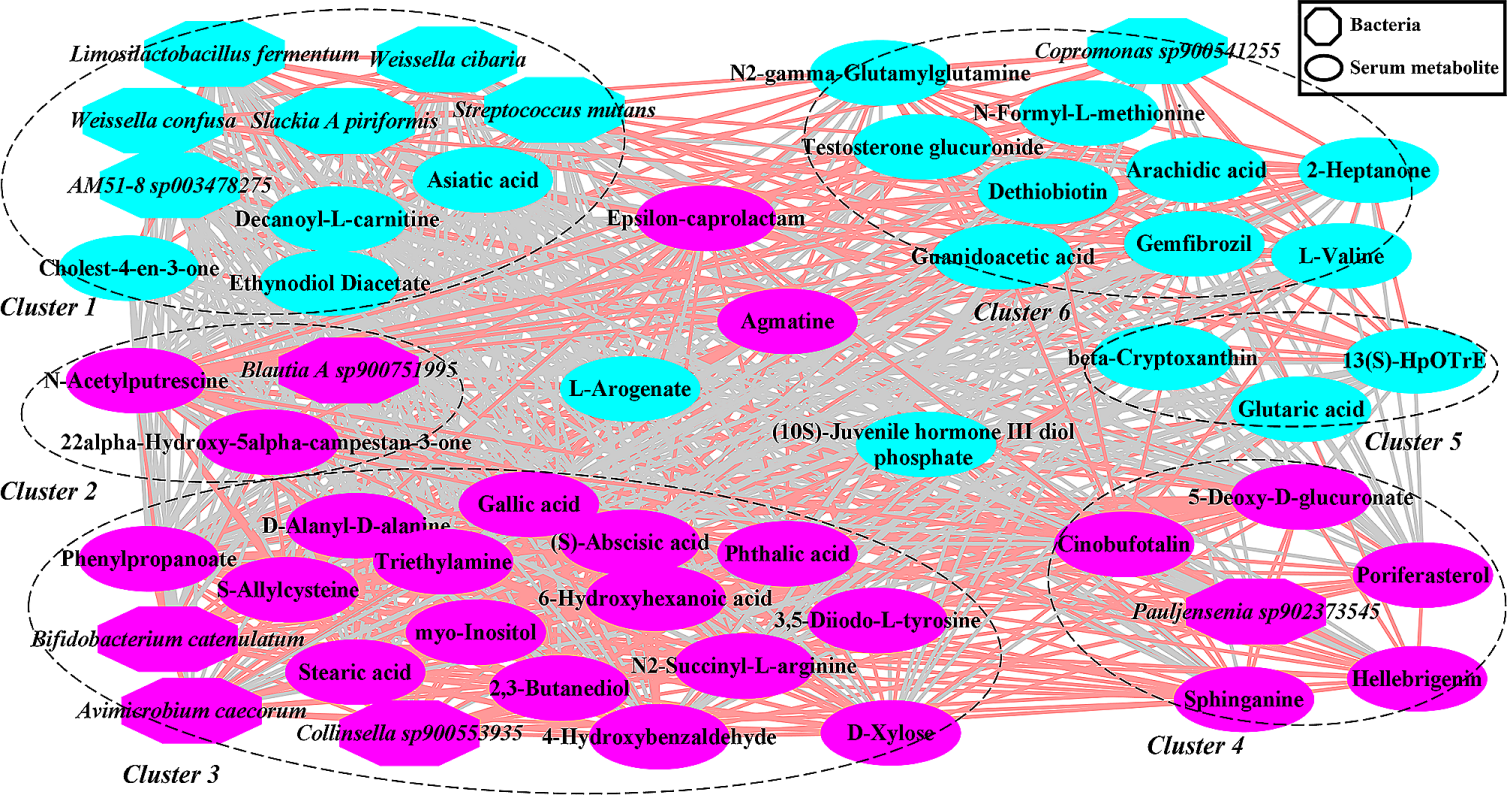
A co-occurrence network constructed with bacterial species and fecal metabolites showing different abundances between severe preeclampsia and healthy controls. Purple and blue dots indicate the severe preeclampsia and healthy control groups, respectively. Edges between nodes indicate Spearman’s negative (light gray) or positive (light red) correlation

We further explored the potential correlations between differential bacterial species and fecal metabolites by co-occurrence network analysis. Strong and broad co-occurring relationships were observed between each other (Fig. 6). The differential bacterial species and fecal metabolites were mainly aggregated into six clusters in this network. The bacterial species belonging to cluster 1 were enriched in healthy pregnant women. The bacterial species enriched in severe preeclampsia were mainly included in cluster 3. The metabolites having higher abundances in healthy controls were primarily included in clusters 1, 5 and 6, while clusters 2, 3, and 4 comprised the metabolites having higher abundances in severe preeclampsia. Interesting, *Limosilactobacillus fermentum* was significantly and positively correlated with the bacterial species in cluster 1.

### Multiple markers associated with the phenotypes of severe preeclampsia

**Fig. 7:**
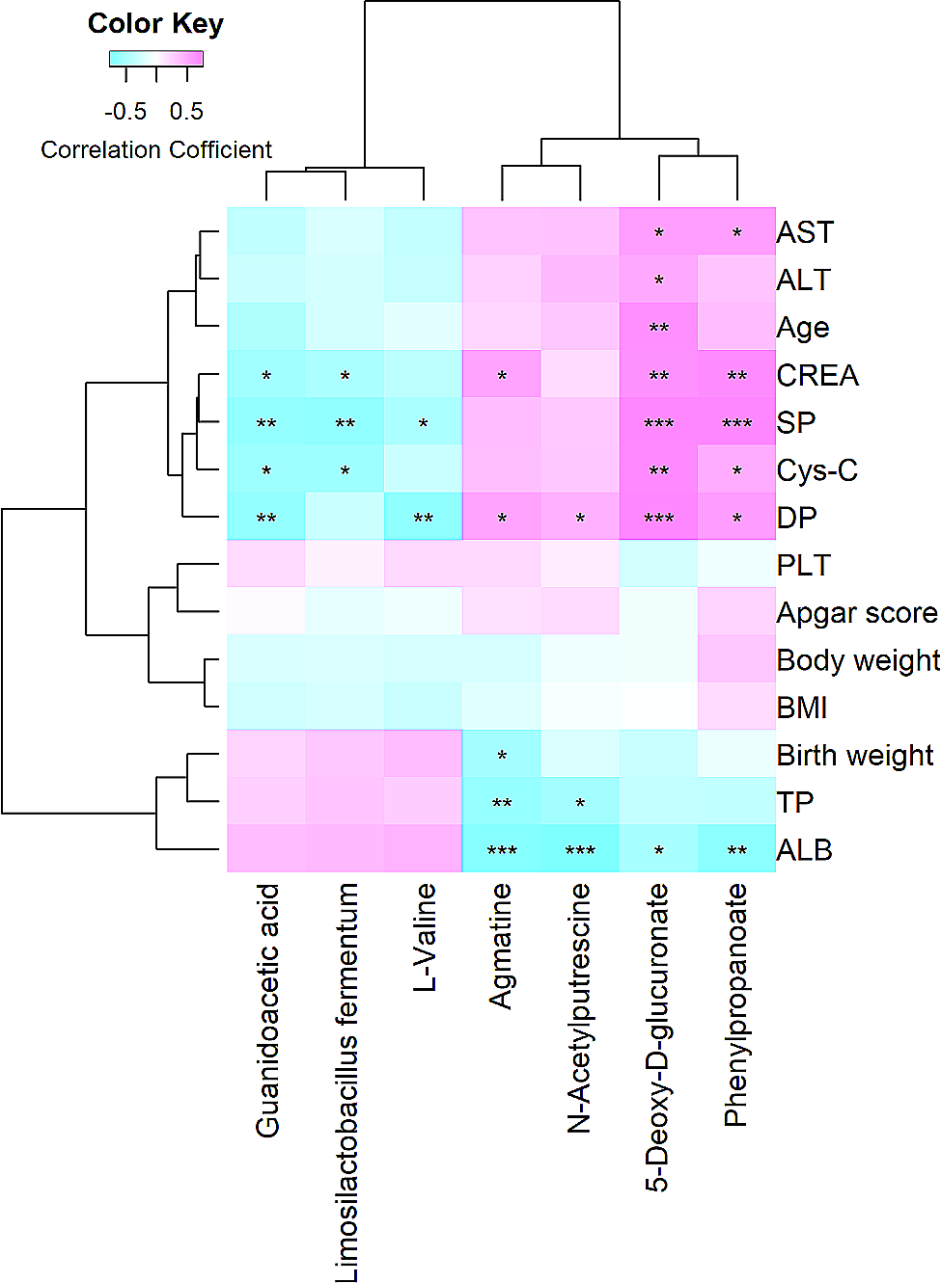
The relationships of altered bacterial species and fecal metabolite features with the 14 phenotypes of severe preeclampsia. The *X*-axis represents differential bacterial species and fecal metabolites. The *Y*-axis represents the 14 phenotypes of severe preeclampsia. * *P* < 0.05, ** *P* < 0.01, and *** *P* < 0.001

We also analyzed the relationship between discriminative bacterial species and phenotypes and discriminative fecal metabolites and phenotypes using the Spearman correlation analysis. The results indicated that seven markers were identified to be significantly associated with several phenotypes (Fig. 7). For example, *Limosilactobacillus fermentum* was negatively and significantly correlated with SP (*r* = -0.64, *P* = 3.86E-03), CREA (*r* = -0.50, *P* = 3.41E-02), and Cys-C (*r* = -0.64, *P* = 3.86E-03). Phenylpropanoate was positively and significantly correlated with SP (*r* = 0.71, *P* = 9.70E-04), DP (*r* = 0.59, *P* = 1.08E-02), AST (*r* = 0.57, *P* = 1.29E-02), CREA (*r* = 0.67, *P* = 2.22E-03), and Cys-C (*r* = 0.50, *P* = 4.20E-02), while ALB was negatively and significantly correlated with phenylpropanoate (*r* = -0.68, *P* = 1.93E-03).

## Discussion

In this study, we compared the bacterial composition of the gut microbiome between severe preeclampsia and healthy controls by metagenomic sequencing and identified bacterial species associated with severe preeclampsia. We identified and independently validated *Limosilactobacillus fermentum* which could distinguish severe preeclampsia from healthy controls with high accuracy. Moreover, we outlined interaction networks of differential bacterial species and differential fecal metabolites. We found that the changes in gut microbiome were correlated with the shifts of fecal metabolome and should result in severe preeclampsia. Our findings lay the foundation for understanding the relationships between the composition and functional capacity of the gut microbiome with severe preeclampsia.

The bacterial α-diversity analysis revealed no significant differences in the Chao, Shannon, and Invsimpson indexes between individuals with severe preeclampsia and healthy controls. Lin et al. also found that species richness in the vaginal microbiota did not differ significantly between cases with severe preeclampsia and controls [34]. However, both Shannon (*P* = 0.001) and Gini-Simpson (*P* < 0.001) indices were higher in cases with severe preeclampsia through 16 S ribosomal RNA gene sequencing of the V3–V4 region [34]. They also found that *Prevotella bivia* in vaginal microbiota, which is tightly regulated by BMI, may be involved in the pathogenesis of severe preeclampsia [34]. The present study also illustrates that the gut microbiota from the healthy controls was substantially different from that of severe preeclampsia, particularly about the higher proportion of Lactobacillaceae detected in fecal microbiota through metagenomic sequencing. Lactobacillaceae can be found in various parts of the human body, including the gastrointestinal tract, reproductive, and urinary systems [35]. A significant proportion of Lactobacillaceae can protect healthy women’s urogenital tract from pathogenic infections [36, 37]. Furthermore, *Limosilactobacillus fermentum* was the only bacterial species showing different abundances between severe preeclampsia and healthy controls through random forest and LEfSe analyses. Our results suggest that the *Limosilactobacillus fermentum* was a key bacterial biomarker related to severe preeclampsia. *Limosilactobacillus fermentum* ferments carbohydrates to produce lactic acid, ethanol/acetic acid, and carbon dioxide as by-products [38]. Several studies have indicated that the effectiveness of administering *Limosilactobacillus fermentum* helps in positively altering the host antioxidant system, which leads to the improvement of various systemic disease phenotypes. This has been observed in both in vitro and experimental studies, as well as in some clinical trials [39, 40]. In the male offspring of female mice fed with a high-fat high-cholesterol diet, the application of fermented *Limosilactobacillus fermentum* reduced systolic and diastolic blood pressure, and mean blood pressure levels, as well as alleviated renal function damage and oxidative stress along the intestinal renal axis [41]. Furthermore, functional capacity analysis revealed that *Limosilactobacillus fermentum* was significantly and positively associated with KEGG pathways related to flagellar assembly. Flagella are a class of important organelles that protrude from the surface of eukaryotic cells and play important roles in cell movement, embryonic development, and signal transduction [42].

Fecal metabolome analysis found that the concentration of Guanidoacetic acid and L-Valine, 5-Deoxy-D-glucuronate, Phenylpropanoate, Agmatine, and N-Acetylputrescine were enriched in healthy pregnant women and severe preeclampsia, respectively. Guanidoacetic acid is an amino acid-like compound that, although known for a long time, is a relatively unexplored human metabolite [43]. Guanidoacetic acid deficiency has been found in many pathologies, ranging from very rare creatine deficiency syndrome and urea cycle defects to more common diseases, including trauma, neurological disorders, and chronic kidney disease [43]. Exogenous Guanidoacetic acid can stimulate hormonal release and neural regulation, alter the metabolic utilization of amino acids (e.g., arginine), and act as an oxidant-antioxidant tuner [44]. Agmatine, an endogenous polyamine derived from L-arginine, elicits tremendous multimodal neuromodulant properties [45]. L-arginine supplementation can enhance anti-infectious, anti-oxidative responses, and immunity; ameliorate metabolic syndromes (including hypertension, obesity, and dyslipidemia); and treat individuals with preeclampsia, muscular dystrophy, and sickle cell disease [46]. Of note, Phenylpropanoate was negatively and significantly correlated with *Limosilactobacillus fermentum* (*P* < 0.001) and could be used to distinguish severe preeclampsia from healthy pregnant women with robust and high diagnostic accuracy of the area under the curve (AUC) 98.57%. Furthermore, the differential metabolites were significantly enriched in the KEGG pathways of arginine and proline metabolism. Arginine and proline are related to immune system regulation and intracellular redox levels [47]. Many studies have found that arginine and proline metabolism contribute to increased inflammatory reactions, uremic toxins, and oxidative stress [48–50]. Especially, Wang et al. revealed that gut dysbiosis in chronic kidney disease was closely related to disordered arginine and proline metabolism [51]. In this study, we also found that *Limosilactobacillus fermentum* was significantly correlated with the metabolites related to arginine and proline metabolism. This suggests that *Limosilactobacillus fermentum* may improve severe preeclampsia through arginine and proline metabolism. Therefore, from the clinical point of view, *Limosilactobacillus fermentum* can be used as a diagnostic biomarker of severe preeclampsia and can be developed as a probiotic that could potentially provide a treatment strategy for severe preeclampsia.

This study has several limitations. The number of samples was relatively small, the results were established based on association studies, and the causality and underlying mechanisms have not been elucidated. These gaps should be addressed in future studies.

## Conclusions

In summary, we found that the composition and functional capacity of fecal microbiome, and fecal metabolites were significantly changed between severe preeclampsia and healthy controls. *Limosilactobacillus fermentum* was identified as a key bacterial species associated with severe preeclampsia; it was significantly correlated with the changes in the functional capacity of flagellar assembly in the gut microbiome. Untargeted fecal metabolome analysis identified that the concentration of Phenylpropanoate and Agmatine were associated with severe preeclampsia and correlated with the abundance of *Limosilactobacillus fermentum* in the gut. Our findings suggest that changes in metabolites, possibly due to gut microbes such as *Limosilactobacillus fermentum*, can contribute to the development of severe preeclampsia. This study provides important insights into the interaction between the gut microbiome and metabolites and offers a theoretical basis for improving severe preeclampsia by modulating the gut microbiome.

### Electronic supplementary material

Below is the link to the electronic supplementary material.

Supplementary Material 1

## Data Availability

The sequencing data of metagenomic sequencing were submitted to China National GeneBank DataBase (CNGBdb) with accession code: CNP0005271.

## Abbreviations

AUC: area under the curve
ALT: Serum alanine aminotransferase
AST: Aspartate aminotransferase
bp: base-pairs
FDR: false discovery rate
ISSHP: International Society for the Study of Hypertension in Pregnancy
KEGG: Kyoto Encyclopedia of Genes and Genomes
KO: KEGG Orthology
LDA: linear discriminant analysis
LEfSe: linear discriminant analysis effect size
NCBI: National Center for Biotechnology Information
NR: non-redundant
ORF: open reading frames
PLS-DA: Partial least squares-discriminant analysis
qPCR: quantitative polymerase chain reaction
QC: quality control
RSD: relative standard deviation
SVR: support vector regression
SparCC: Sparse Correlations for Compositional

## References

[1] Mol BWJ, Roberts CT, Thangaratinam S, Magee LA, de Groot CJM, Hofmeyr GJ (2016). Pre-eclampsia. Lancet.

[2] Li X, Milosavljevic A, Elsea SH, Wang CC, Scaglia F, Syngelaki A, Nicolaides KH, Poon LC (2021). Effective aspirin treatment of women at risk for Preeclampsia Delays the metabolic clock of Gestation. Hypertension.

[3] Say L, Chou D, Gemmill A, Tuncalp O, Moller AB, Daniels J, Gulmezoglu AM, Temmerman M, Alkema L (2014). Global causes of maternal death: a WHO systematic analysis. Lancet Glob Health.

[4] Ackerman CM, Platner MH, Spatz ES, Illuzzi JL, Xu X, Campbell KH, Smith GN, Paidas MJ, Lipkind HS. Severe cardiovascular morbidity in women with hypertensive diseases during delivery hospitalization. Am J Obstet Gynecol. 2019; 220:582 e581-582 e511. 10.1016/j.ajog.2019.02.010 30742823

[5] Hitti J, Sienas L, Walker S, Benedetti TJ, Easterling T. Contribution of hypertension to severe maternal morbidity. Am J Obstet Gynecol. 2018;219:e405401–7. 10.1016/j.ajog.2018.07.002 30012335

[6] Nelson DB, Ziadie MS, McIntire DD, Rogers BB, Leveno KJ. Placental pathology suggesting that preeclampsia is more than one disease. Am J Obstet Gynecol. 2014;210:e6661–67. 10.1016/j.ajog.2013.09.010 24036400

[7] Udenze I, Amadi C, Awolola N, Makwe CC (2015). The role of cytokines as inflammatory mediators in preeclampsia. Pan Afr Med J.

[8] Zuniga FA, Ormazabal V, Gutierrez N, Aguilera V, Radojkovic C, Veas C, Escudero C, Lamperti L, Aguayo C. Role of lectin-like oxidized low density lipoprotein-1 in fetoplacental vascular dysfunction in preeclampsia. Biomed Res Int. 2014; 2014:353616. 10.1155/2014/353616 PMC4109675 25110674

[9] Kang Y, Cai Y (2018). Gut microbiota and hypertension: from pathogenesis to new therapeutic strategies. Clin Res Hepatol Gastroenterol.

[10] Maifeld A, Bartolomaeus H, Lober U, Avery EG, Steckhan N, Marko L, Wilck N, Hamad I, Susnjar U, Mahler A et al. Fasting alters the gut microbiome reducing blood pressure and body weight in metabolic syndrome patients. Nat Commun. 2021; 12:1970. 10.1038/s41467-021-22097-0 PMC8010079 33785752

[11] Wilck N, Matus MG, Kearney SM, Olesen SW, Forslund K, Bartolomaeus H, Haase S, Mahler A, Balogh A, Marko L (2017). Salt-responsive gut commensal modulates T(H)17 axis and disease. Nature.

[12] Yan X, Jin J, Su X, Yin X, Gao J, Wang X, Zhang S, Bu P, Wang M, Zhang Y (2020). Intestinal Flora modulates blood pressure by regulating the synthesis of intestinal-derived corticosterone in High Salt-Induced Hypertension. Circ Res.

[13] Liu J, Yang H, Yin Z, Jiang X, Zhong H, Qiu D, Zhu F, Li R (2017). Remodeling of the gut microbiota and structural shifts in Preeclampsia patients in South China. Eur J Clin Microbiol Infect Dis.

[14] Lv LJ, Li SH, Li SC, Zhong ZC, Duan HL, Tian C, Li H, He W, Chen MC, He TW (2019). Early-Onset Preeclampsia is Associated with gut microbial alterations in Antepartum and Postpartum women. Front Cell Infect Microbiol.

[15] Wang J, Gu X, Yang J, Wei Y, Zhao Y (2019). Gut microbiota dysbiosis and increased plasma LPS and TMAO levels in patients with Preeclampsia. Front Cell Infect Microbiol.

[16] Brantsaeter AL, Myhre R, Haugen M, Myking S, Sengpiel V, Magnus P, Jacobsson B, Meltzer HM (2011). Intake of probiotic food and risk of preeclampsia in primiparous women: the Norwegian mother and child cohort study. Am J Epidemiol.

[17] Jin J, Gao L, Zou X, Zhang Y, Zheng Z, Zhang X, Li J, Tian Z, Wang X, Gu J (2022). Gut dysbiosis promotes Preeclampsia by regulating macrophages and trophoblasts. Circ Res.

[18] Benschop L, Duvekot JJ, Versmissen J, van Broekhoven V, Steegers EAP (2018). Roeters Van Lennep JE. Blood pressure Profile 1 year after severe Preeclampsia. Hypertension.

[19] Bulletins, –, Obstetrics ACoP (2002). ACOG practice bulletin. Diagnosis and management of preeclampsia and eclampsia. Number 33, January 2002. Obstet Gynecol.

[20] Martin M. Cutadapt removes adapter sequences from high-throughput sequencing reads. EMBnet J. 2011; 17.

[21] Chen S, Zhou Y, Chen Y, Gu J (2018). Fastp: an ultra-fast all-in-one FASTQ preprocessor. Bioinformatics.

[22] Rotmistrovsky K, Agarwala R, BMTagger. Best Match Tagger for removing human reads from metagenomics datasets. NCBI/NLM. 2011.

[23] Wood DE, Lu J, Langmead B (2019). Improved metagenomic analysis with Kraken 2. Genome Biol.

[24] Li D, Liu CM, Luo R, Sadakane K, Lam TW (2015). MEGAHIT: an ultra-fast single-node solution for large and complex metagenomics assembly via succinct de bruijn graph. Bioinformatics.

[25] Steinegger M, Soding J (2017). MMseqs2 enables sensitive protein sequence searching for the analysis of massive data sets. Nat Biotechnol.

[26] Zhu W, Lomsadze A, Borodovsky M (2010). Ab initio gene identification in metagenomic sequences. Nucleic Acids Res.

[27] Patro R, Duggal G, Kingsford C, Salmon. Accurate, Versatile and Ultrafast Quantification from RNA-seq Data using Lightweight-Alignment. bioRxiv. 2015.

[28] Zelena E, Dunn WB, Broadhurst D, Francis-McIntyre S, Carroll KM, Begley P, O’Hagan S, Knowles JD, Halsall A, Consortium H (2009). Development of a robust and repeatable UPLC-MS method for the long-term metabolomic study of human serum. Anal Chem.

[29] Want EJ, Masson P, Michopoulos F, Wilson ID, Theodoridis G, Plumb RS, Shockcor J, Loftus N, Holmes E, Nicholson JK (2013). Global metabolic profiling of animal and human tissues via UPLC-MS. Nat Protoc.

[30] Rasmussen JA, Villumsen KR, Ernst M, Hansen M, Forberg T, Gopalakrishnan S, Gilbert MTP, Bojesen AM, Kristiansen K, Limborg MT (2022). A multi-omics approach unravels metagenomic and metabolic alterations of a probiotic and synbiotic additive in rainbow trout (Oncorhynchus mykiss). Microbiome.

[31] Navarro-Reig M, Jaumot J, Garcia-Reiriz A, Tauler R (2015). Evaluation of changes induced in rice metabolome by cd and Cu exposure using LC-MS with XCMS and MCR-ALS data analysis strategies. Anal Bioanal Chem.

[32] Ibba M, Soll D (2000). Aminoacyl-tRNA synthesis. Annu Rev Biochem.

[33] Ye JZ, Lin XM, Cheng ZX, Su YB, Li WX, Ali FM, Zheng J, Peng B (2018). Identification and efficacy of glycine, serine and threonine metabolism in potentiating kanamycin-mediated killing of Edwardsiella piscicida. J Proteom.

[34] Lin CY, Lin CY, Yeh YM, Yang LY, Lee YS, Chao A, Chin CY, Chao AS, Yang CY (2020). Severe preeclampsia is associated with a higher relative abundance of Prevotella bivia in the vaginal microbiota. Sci Rep.

[35] Gonzalez-Lozano E, Garcia-Garcia J, Galvez J, Hidalgo-Garcia L, Rodriguez-Nogales A, Rodriguez-Cabezas ME, Sanchez M. Novel Horizons in Postbiotics: Lactobacillaceae Extracellular vesicles and their applications in Health and Disease. Nutrients. 2022; 14. 10.3390/nu14245296 PMC9782203 36558455

[36] De Gregorio PR, Tomas MSJ, Terraf MCL, Nader-Macias MEF (2014). In vitro and in vivo effects of beneficial vaginal lactobacilli on pathogens responsible for urogenital tract infections. J Med Microbiol.

[37] Zhang Z, Lv J, Pan L, Zhang Y (2018). Roles and applications of probiotic Lactobacillus strains. Appl Microbiol Biotechnol.

[38] Dempsey E, Corr SC (2022). Lactobacillus spp. for Gastrointestinal Health: current and future perspectives. Front Immunol.

[39] Paulino do Nascimento LC, Lacerda DC, Ferreira DJS, de Souza EL, de Brito Alves JL (2022). Limosilactobacillus Fermentum, current evidence on the antioxidant properties and opportunities to be exploited as a probiotic microorganism. Probiotics Antimicrob Proteins.

[40] Lacerda DC, Trindade da Costa PC, Pontes PB, Carneiro Dos Santos LA, Cruz Neto JPR, Silva Luis CC, de Sousa Brito VP, de Brito Alves JL (2022). Potential role of limosilactobacillus fermentum as a probiotic with anti-diabetic properties: a review. World J Diabetes.

[41] do Nascimento LCP, de Souza EL, de Luna Freire MO, de Andrade Braga V, de Albuqeurque TMR, Lagranha CJ, de Brito Alves JL (2022). Limosilactobacillus Fermentum prevents gut-kidney oxidative damage and the rise in blood pressure in male rat offspring exposed to a maternal high-fat diet. J Dev Orig Health Dis.

[42] Nachury MV, Mick DU (2019). Establishing and regulating the composition of cilia for signal transduction. Nat Rev Mol Cell Biol.

[43] Ostojic SM, Ratgeber L, Olah A, Betlehem J, Acs P (2020). Guanidinoacetic acid deficiency: a new entity in clinical medicine?. Int J Med Sci.

[44] Ostojic SM (2015). Advanced physiological roles of guanidinoacetic acid. Eur J Nutr.

[45] Saha P, Panda S, Holkar A, Vashishth R, Rana SS, Arumugam M, Ashraf GM, Haque S, Ahmad F (2023). Neuroprotection by agmatine: possible involvement of the gut microbiome?. Ageing Res Rev.

[46] Wu G, Meininger CJ, McNeal CJ, Bazer FW, Rhoads JM (2021). Role of L-Arginine in Nitric Oxide Synthesis and Health in humans. Adv Exp Med Biol.

[47] Song G, Gan Q, Qi W, Wang Y, Xu M, Li Y. Fructose Stimulated Colonic Arginine and Proline Metabolism Dysbiosis, altered Microbiota and aggravated intestinal barrier dysfunction in DSS-Induced colitis rats. Nutrients. 2023; 15. 10.3390/nu15030782 PMC9921751 36771488

[48] Wang T, Fu X, Chen Q, Patra JK, Wang D, Wang Z, Gai Z. Arachidonic acid metabolism and kidney inflammation. Int J Mol Sci. 2019; 20. 10.3390/ijms20153683 PMC6695795 31357612

[49] Li X, Wang Y, Gao M, Bao B, Cao Y, Cheng F, Zhang L, Li Z, Shan J, Yao W (2022). Metabolomics-driven of relationships among kidney, bone marrow and bone of rats with postmenopausal osteoporosis. Bone.

[50] Aw TY (2005). Intestinal glutathione: determinant of mucosal peroxide transport, metabolism, and oxidative susceptibility. Toxicol Appl Pharmacol.

[51] Wang H, Ainiwaer A, Song Y, Qin L, Peng A, Bao H, Qin H (2023). Perturbed gut microbiome and fecal and serum metabolomes are associated with chronic kidney disease severity. Microbiome.

