# Supplementary material for: Landscapes of gut bacterial and fecal metabolic signatures and their relationship in severe preeclampsia
Source: J Transl Med. 2024 Apr 17;22:360. doi: 10.1186/s12967-024-05143-5 (PMC11022388; doi:10.1186/s12967-024-05143-5)
Supplement: Supplementary file 1 — Supplementary Material 1 [file 12967_2024_5143_MOESM1_ESM.docx]

**Supporting information**

**Landscapes of gut bacterial and fecal metabolic signatures and their relationship in severe preeclampsia**

*Xianxian Liu^1^, Xiaoming Zeng^1^, Xing Li^3^, Siming Xin^1^, Feng Zhang^1^, Faying Liu^1^, Yang Zeng^1^, Jilin Wu^1^, Yang Zou^1,^*, Xinwei Xiong^2,^**

*^1^**Key Laboratory of Women’s Reproductive Health of Jiangxi Province, Jiangxi Maternal and Child Health Hospital, Nanchang, Jiangxi 330006, China*

*^2^Institute of Biological Technology,* *Nanchang Normal University, Nanchang, Jiangxi 330032, China.*

*^3^Medical Center of Burn Plastic and Wound Repair, The First Affiliated Hospital of Nanchang University, Jiangxi Medical College, Nanchang University，330006,Nanchang, China*

*Corresponding author:

Yang Zou, E-mail: [zouyang81@163.com](mailto:zouyang81@163.com), Key Laboratory of Women’s Reproductive Health of Jiangxi Province, Jiangxi Maternal and Child Health Hospital, Nanchang 330006, China. Phone: 0086 791 86310442, Fax: 0086 791 86310442

Xinwei Xiong, E-mail: [xinweixiong@hotmail.com](mailto:xinweixiong@hotmail.com), Institute of Biological Technology, Nanchang Normal University, Nanchang 330032, China. Phone: 0086-791- 83812115, Fax: 0086-791- 83812115


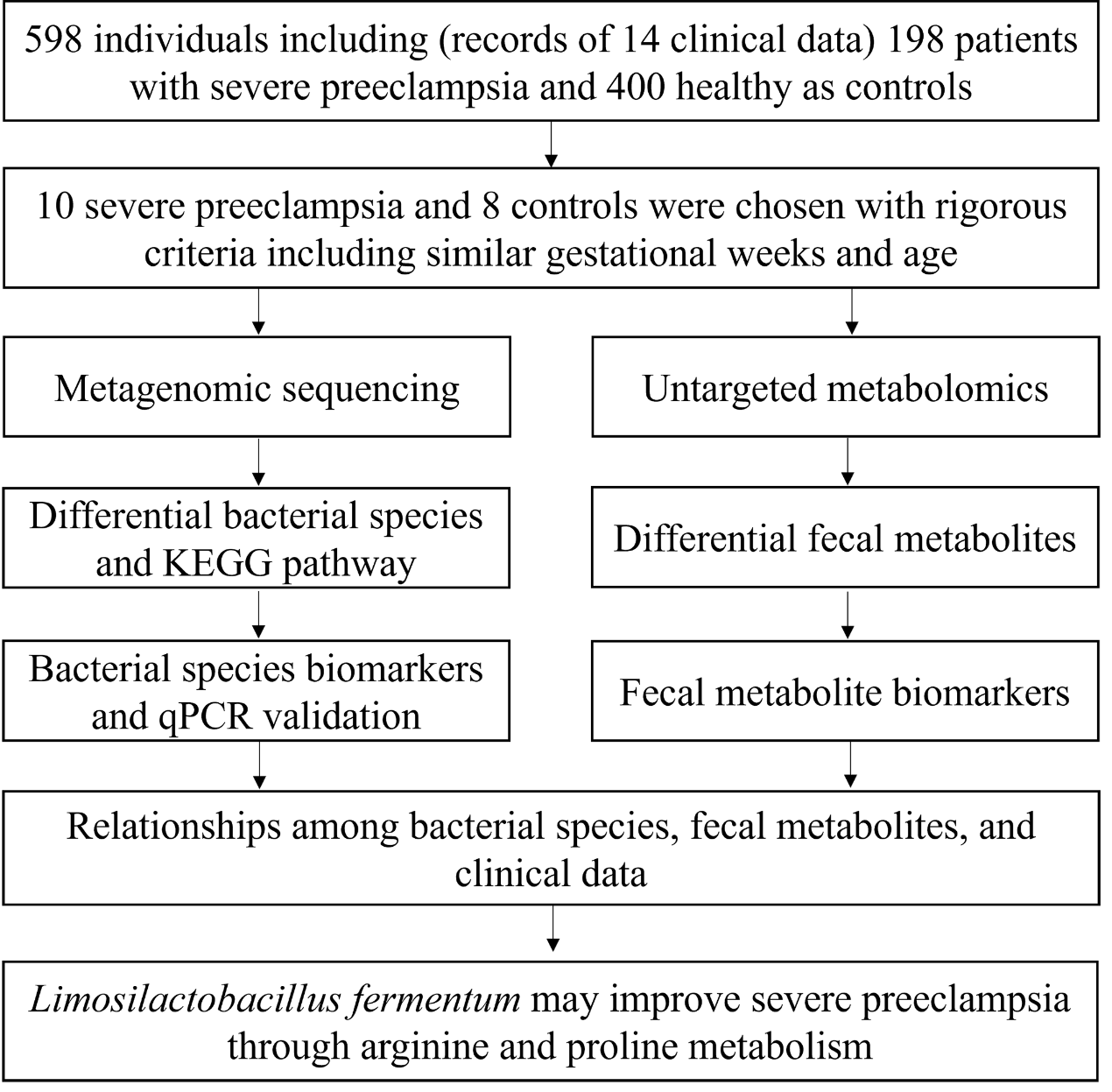


**Figure S1. The overview flow chart of this study.**


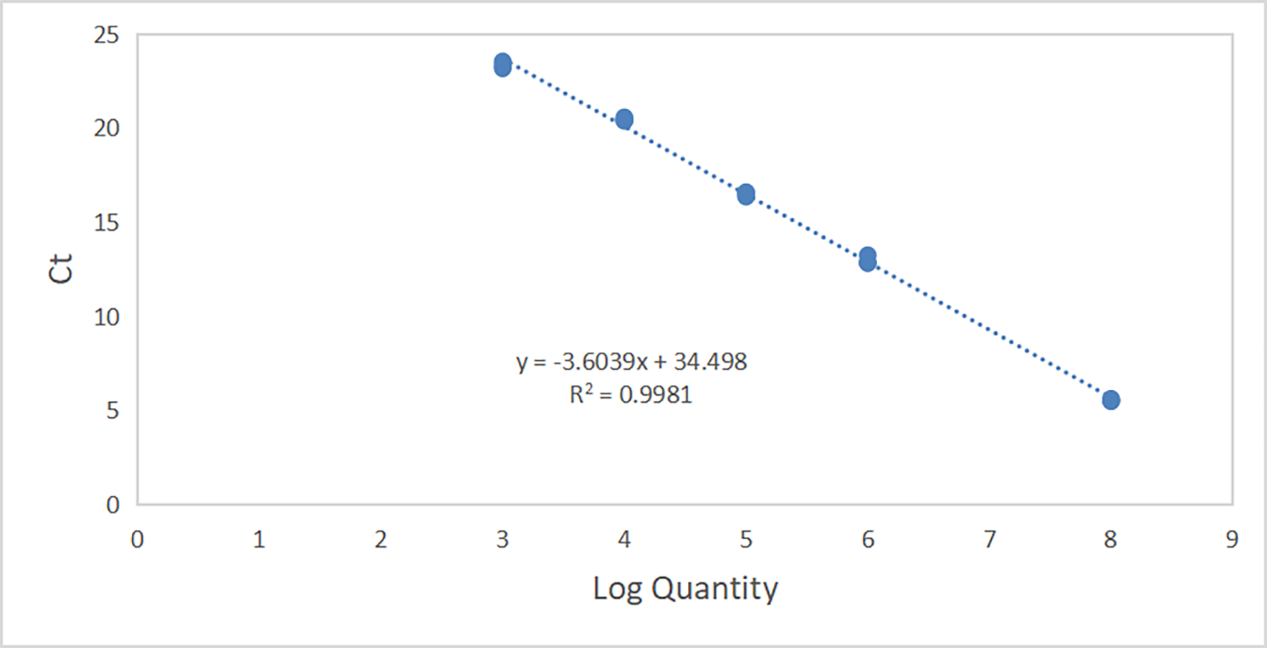


**Figure S2. The standard curve was constructed in 8 of 10-fold diluted plasmids harboring the *16S* gene.**

**Table S1. The sequence assembly analysis for shotgun metagenomic sequencing.**

| **ID** | **Min sequence length** | **Max sequence length** | **Total sequence number** | **N20** | **N50** | **N90** | **Total sequence length** | **Average sequence length** | **GC Number** | **GC content** | **Sequences greater than 1kb** |
| --- | --- | --- | --- | --- | --- | --- | --- | --- | --- | --- | --- |
| PE245 | 200 | 604418 | 146140 | 43252 | 6846 | 613 | 293667308 | 2009 | 147437209 | 0.50 | 47920 |
| PE246 | 200 | 506021 | 132282 | 65010 | 10444 | 649 | 299511001 | 2264 | 146085275 | 0.49 | 43380 |
| PE255 | 200 | 645800 | 121093 | 41646 | 4724 | 596 | 223566490 | 1846 | 112422351 | 0.50 | 40517 |
| PE267 | 200 | 604417 | 123795 | 62157 | 6636 | 609 | 247056063 | 1996 | 121004423 | 0.49 | 39927 |
| PE276 | 200 | 358557 | 200222 | 19539 | 2788 | 531 | 299839031 | 1498 | 146349069 | 0.49 | 61125 |
| PE291 | 200 | 484253 | 184223 | 48375 | 6967 | 576 | 356916879 | 1937 | 174324896 | 0.49 | 53767 |
| PE300 | 200 | 579862 | 231274 | 24838 | 3860 | 547 | 379961280 | 1643 | 192030440 | 0.51 | 69752 |
| PE323 | 200 | 486437 | 153228 | 38574 | 5872 | 557 | 275641431 | 1799 | 138528685 | 0.50 | 44218 |
| PE357 | 200 | 668020 | 106134 | 33769 | 5177 | 640 | 212313659 | 2000 | 101738504 | 0.48 | 39496 |
| PE364 | 200 | 496343 | 85082 | 70161 | 11510 | 662 | 199076907 | 2340 | 92582433 | 0.47 | 28066 |
| N73 | 200 | 595989 | 217909 | 26390 | 4062 | 545 | 355355742 | 1631 | 174039971 | 0.49 | 63085 |
| N74 | 200 | 440029 | 93623 | 69269 | 11391 | 750 | 245235485 | 2619 | 117319133 | 0.48 | 35078 |
| N75 | 200 | 474714 | 134536 | 33541 | 3605 | 515 | 205341584 | 1526 | 99110959 | 0.48 | 34350 |
| N77 | 200 | 741039 | 149372 | 35528 | 4635 | 578 | 261736689 | 1752 | 124706332 | 0.48 | 46221 |
| N78 | 200 | 427193 | 187909 | 28058 | 4251 | 560 | 318966902 | 1697 | 158651151 | 0.50 | 57344 |
| N79 | 200 | 524413 | 143720 | 51724 | 6176 | 594 | 282027062 | 1962 | 138599510 | 0.49 | 45962 |
| N81 | 200 | 679219 | 88706 | 49862 | 7187 | 737 | 212236421 | 2393 | 101112020 | 0.48 | 36150 |
| N82 | 200 | 537140 | 119138 | 60949 | 10163 | 626 | 258293618 | 2168 | 123881747 | 0.48 | 37100 |

**Table S2. The differential metabolite features between severe preeclampsia and healthy controls through Wilcoxon analysis.**

| **Name** | ***P*** |
| --- | --- |
| 5-Deoxy-D-glucuronate | 3.01E-05 |
| Phenylpropanoate | 1.75E-04 |
| Guanidoacetic acid | 5.13E-04 |
| 2,3-Butanediol | 7.13E-04 |
| D-Alanyl-D-alanine | 1.66E-03 |
| myo-Inositol | 1.74E-03 |
| 6-Hydroxyhexanoic acid | 2.14E-03 |
| L-Arogenate | 2.28E-03 |
| L-Valine | 3.23E-03 |
| S-Allylcysteine | 5.42E-03 |
| N-Acetylputrescine | 5.66E-03 |
| Asiatic acid | 7.80E-03 |
| Gemfibrozil | 1.16E-02 |
| beta-Cryptoxanthin | 1.20E-02 |
| (10S)-Juvenile hormone III diol phosphate | 1.38E-02 |
| Gallic acid | 1.38E-02 |
| N2-gamma-Glutamylglutamine | 1.43E-02 |
| Stearic acid | 1.52E-02 |
| Ethynodiol Diacetate | 1.64E-02 |
| Dethiobiotin | 1.81E-02 |
| D-Xylose | 1.86E-02 |
| N2-Succinyl-L-arginine | 2.61E-02 |
| (S)-Abscisic acid | 2.69E-02 |
| Epsilon-caprolactam | 2.74E-02 |
| 13(S)-HpOTrE | 3.11E-02 |
| 2-Heptanone | 4.34E-02 |
| Phthalic acid | 4.35E-02 |
| Agmatine | 4.37E-02 |
| 4-Hydroxybenzaldehyde | 4.52E-02 |
| Triethylamine | 4.67E-02 |
| Poriferasterol | 4.75E-02 |

**Table S3. The differential metabolite features between severe preeclampsia and healthy controls through LASSO regression analysis.**

| **Name** | ***P*** |
| --- | --- |
| Guanidoacetic acid | -6.94E-08 |
| N-Formyl-L-methionine | -1.30E-08 |
| L-Valine | -8.39E-09 |
| Testosterone glucuronide | -4.58E-09 |
| Decanoyl-L-carnitine | -2.77E-09 |
| Guanosine | -3.86E-11 |
| D-Fructose | -1.29E-12 |
| N-Acetylputrescine | 2.29E-10 |
| Hellebrigenin | 6.66E-10 |
| Sphinganine | 7.82E-10 |
| 3,5-Diiodo-L-tyrosine | 1.56E-09 |
| Cinobufotalin | 2.16E-09 |
| 5-Deoxy-D-glucuronate | 5.18E-09 |
| Epsilon-caprolactam | 6.37E-09 |
| 22alpha-Hydroxy-5alpha-campestan-3-one | 6.93E-09 |
| Phenylpropanoate | 9.30E-09 |
| Agmatine | 9.81E-09 |
